# [Retracted] Hesperidin exhibits *in vitro* and *in vivo* antitumor effects in human osteosarcoma MG‑63 cells and xenograft mice models via inhibition of cell migration and invasion, cell cycle arrest and induction of mitochondrial‑mediated apoptosis

**DOI:** 10.3892/ol.2023.13703

**Published:** 2023-02-06

**Authors:** Guang-Yu Du, Sheng-Wei He, Lu Zhang, Chuan-Xiu Sun, Li-Dong Mi, Zue-Gang Sun

Oncol Lett 16: 6299–6306, 2018; DOI: 10.3892/ol.2018.9439

Following the publication of this paper, it was drawn to the Editors’ attention by a concerned reader that Figs. 3, 5, 6, 7 and 9 in this paper all contained anomalous features that could not readily be explained by chance alone or through the coincidental placement of data. Consequently, the Editor of *Oncology Letters* has determined that this paper should be retracted from the Journal. The authors were asked for an explanation to account for these concerns, but the Editorial Office did not receive a reply. The Editor apologizes to the readership for any inconvenience caused.

